# Positron emission tomography in the COVID-19 pandemic era

**DOI:** 10.1007/s00259-021-05347-7

**Published:** 2021-05-19

**Authors:** Chentao Jin, Xiaoyun Luo, Shufang Qian, Kai Zhang, Yuanxue Gao, Rui Zhou, Peili Cen, Zhoujiao Xu, Hong Zhang, Mei Tian

**Affiliations:** 1 Department of Nuclear Medicine and PET Center, The Second Affiliated Hospital of Zhejiang University School of Medicine, 88 Jiefang Road, Zhejiang, 310009 Hangzhou China; 2 Institute of Nuclear Medicine and Molecular Imaging of Zhejiang University, Hangzhou, China; 3 Key Laboratory of Medical Molecular Imaging of Zhejiang Province, Hangzhou, China; 4 Laboratory for Pathophysiological and Health Science, RIKEN Center for Biosystems Dynamics Research, Kobe, Hyogo Japan; 5 College of Biomedical Engineering & Instrument Science, Zhejiang University, Hangzhou, China; 6 Key Laboratory for Biomedical Engineering of Ministry of Education, Zhejiang University, Hangzhou, China

**Keywords:** COVID-19, SARS-CoV-2, Positron emission tomography (PET), Molecular imaging

## Abstract

Coronavirus disease 2019 (COVID-19) has become a major public health problem worldwide since its outbreak in 2019. Currently, the spread of COVID-19 is far from over, and various complications have roused increasing awareness of the public, calling for novel techniques to aid at diagnosis and treatment. Based on the principle of molecular imaging, positron emission tomography (PET) is expected to offer pathophysiological alternations of COVID-19 in the molecular/cellular perspectives and facilitate the clinical management of patients. A number of PET-related cases and research have been reported on COVID-19 over the past one year. This article reviews the current studies of PET in the diagnosis and treatment of COVID-19, and discusses potential applications of PET in the development of management strategy for COVID-19 patients in the pandemic era.

## Introduction

Coronavirus disease 2019 (COVID-19) has become one of the most severe public health problems in the world since the outbreak at the end of 2019, affecting more than two hundred countries and one hundred million people worldwide [1]. Though the pandemic has been under control to some extent in several countries, the global prevalence is still rapidly increasing [2, 3]. A more comprehensive understanding on imaging manifestations of COVID-19 is essential for the identification and early evaluation of the huge patient population. Besides, increasing studies have reported that a growing number of COVID-19 patients, even for those recovered, are facing the threat of various acute or chronic complications, which may lead to a new burden on global health [4, 5]. The formidable challenges facing global health systems raise the unprecedented need for effective approaches to facilitate better strategies of diagnosis and treatment for COVID-19 patients.

**Fig. 1:**
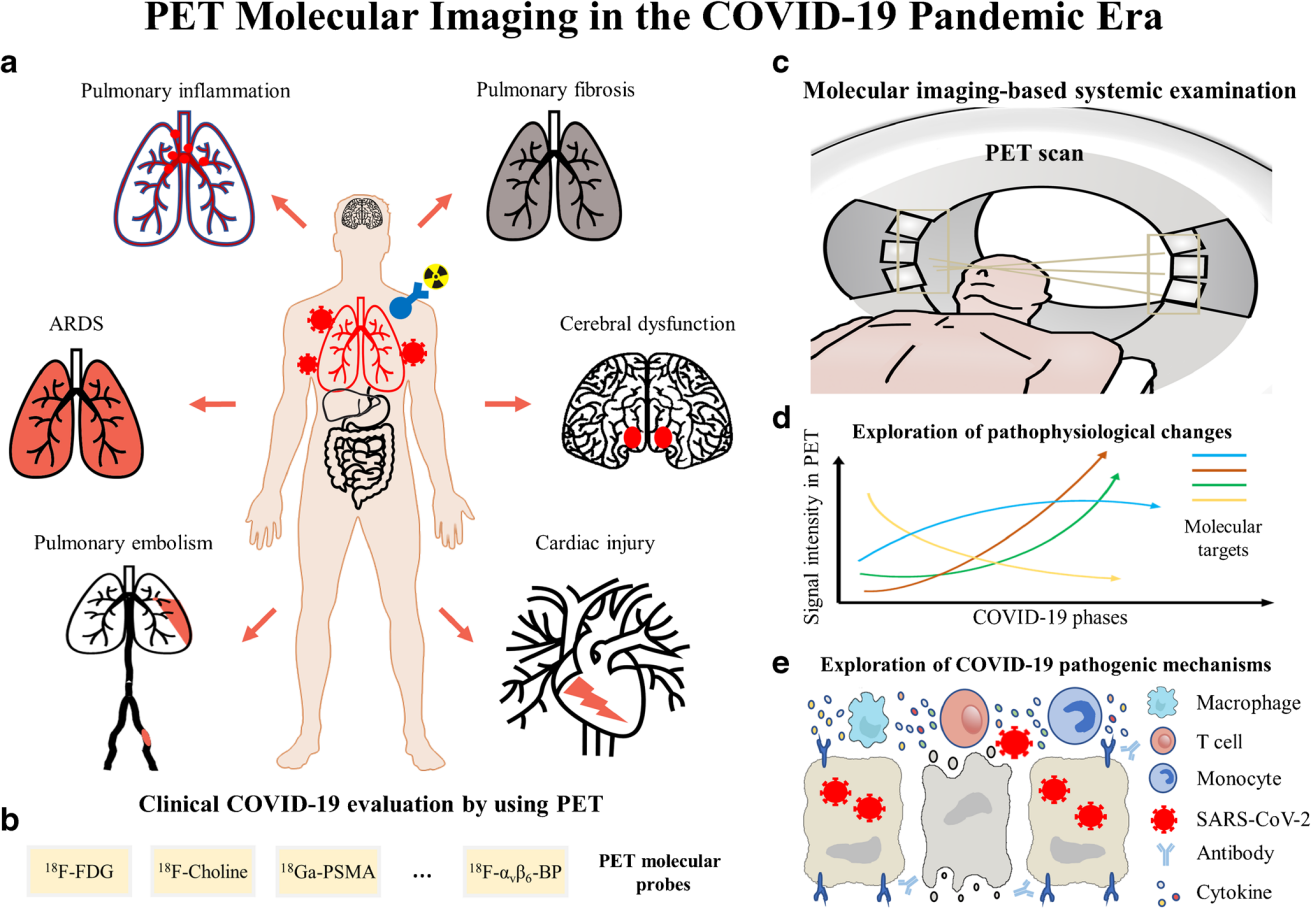
Schematic diagram illustrating the role of PET in the pandemic era. A variety of clinical applications of PET imaging have been reported, facilitating the evaluation of pulmonary inflammation, ARDS, pulmonary embolism, pulmonary fibrosis, cerebral dysfunction, and cardiac injury (**a**), by using various PET molecular probes (**b**). PET enables the whole-body evaluation for COVID-19 patients in molecular perspectives (**c**), helping the exploration of pathophysiological changes of molecular targets in different COVID-19 phases by using specific radiotracers (**d**), as well as the exploration of COVID-19 pathogenic mechanisms through the visualization of molecules involved in the occurrence and development of COVID-19 (**e**)

Positron emission tomography (PET) serves as a representative of molecular imaging modalities and transpathology [6]. By combining with radiopharmaceuticals labeled with positron-emitting radionuclides such as ^11^C, ^13^N, ^18^F, and ^68^Ga, PET enables the visualization of biochemical changes in cellular and molecular perspectives, and thereby shows the great potential to aid in the management of multiple major diseases [7–10]. The application of PET imaging makes it possible to monitor vital pathophysiological alternations of COVID-19 at the molecular level, and thus provides essential guidance for the subsequent diagnosis, evaluation, and treatment of COVID-19 (Fig. 1). Herein, we will review the current studies of PET in the diagnosis and treatment of COVID-19 and discuss potential applications of PET in the development of management strategy for COVID-19 patients in the pandemic era.

## Brief introduction of COVID-19

COVID-19 is a novel acute respiratory infectious disease that was first reported to the World Health Organization (WHO) by Chinese doctors in December 2019 [11], and the causative pathogen of COVID-19 was named Severe Acute Respiratory Syndrome Coronavirus 2 (SARS-CoV-2) [12]. COVID-19 is the third serious Coronavirus outbreak over the last two decades, following Severe Acute Respiratory Syndrome (SARS) and Middle East Respiratory Syndrome (MERS). Interstitial pneumonia is the predominant manifestation of COVID-19, while pathological changes of other organs such as the brain and heart may also occur [13]. Screening for COVID-19 requires a combination of epidemiological risk, clinical manifestations, laboratory tests, and imaging features, while nucleic acid testing, gene sequencing, and serum levels of antibody remain the gold standard for diagnosis [14]. As nucleic acid test has a high false-negative rate, imaging examinations such as chest CT can also provide important evidence for patients’ admission and quarantine in the epidemic areas with high pretest probability, especially where nucleic acid detection kits are in short supply [15]. At present, supportive care is still the main treatment strategy for patients with COVID-19, while recent studies indicated that some other therapies such as dexamethasone could help to reduce mortality [16]. While the general infection fatality rate of COVID-19 was 0.68% according to a meta-analysis, severe complications such as acute respiratory distress syndrome (ARDS) would cause higher mortality, which varied significantly from country to country (e.g., China 69%, Poland 73%, France 19%, Germany 13%) [17, 18]. In addition, with the progress of COVID-19 research, a variety of long-term complications in multiple systems, such as pulmonary fibrosis, delirium, and myocarditis, have been increasingly reported, highlighting a crucial need for further studies [4, 5].

## Nuclear medicine departments in the COVID-19 pandemic era

Both outpatient and inpatient care are massively reduced in most hospitals in order to prevent and control the outbreak, no exception for the department of nuclear medicine [19]. According to an Internet survey concerning multiple nuclear medicine services from the International Atomic Energy Agency, which received answers from 434 physicians from 72 countries, the services of nuclear medicine decreased sharply, by over 50% for diagnostic tests and as much as 45% for radionuclide therapies [20]. Similarly, for another international investigation performed by the Italian Nuclear Medicine Association, results showed declines by over 20% for diagnosis and treatment services caused by the epidemic [21]. Moreover, according to a predicting model established, the COVID-19 may even last until 2025 [22]. In the pandemic era, nuclear medicine workers need to be vigilant throughout service. It is crucially important for guaranteeing the daily work of the nuclear medicine department to develop the monitoring and screening system for both patients and doctors, strengthen personal protection and hygiene measures, and establish emergency plans once new infections appear [23]. Additionally, accompanied with a deeper understanding of the mechanisms for COVID-19 infection, spread, and progression, as well as the continuous improvement of treatment methods and preventive measures, nuclear medicine services are gearing up back to normality [24]. Thus, for a long time in the future, it is essential for nuclear medicine physicians to take advantage of molecular imaging to explore the in-depth mechanisms of COVID-19, develop new methods for diagnosis, treatment, and prevention, so as to help in reducing the burden on world health in the pandemic era.

## PET in the COVID-19 detection

Chest imaging is the priority imaging examination of COVID-19. Compared with common imaging modalities, such as chest X-ray and CT imaging, PET imaging is not considered a routine examination for COVID-19 detection due to its low global availability (especially considering countries not rich), high examination cost, complex scanning procedures, and concomitant risks of virus spreading [25]. However, during the past year, increasing clinical cases of incidental detection of COVID-19 pneumonia by PET imaging have been reported, especially in the oncological whole-body evaluation [26].

**Fig. 2:**
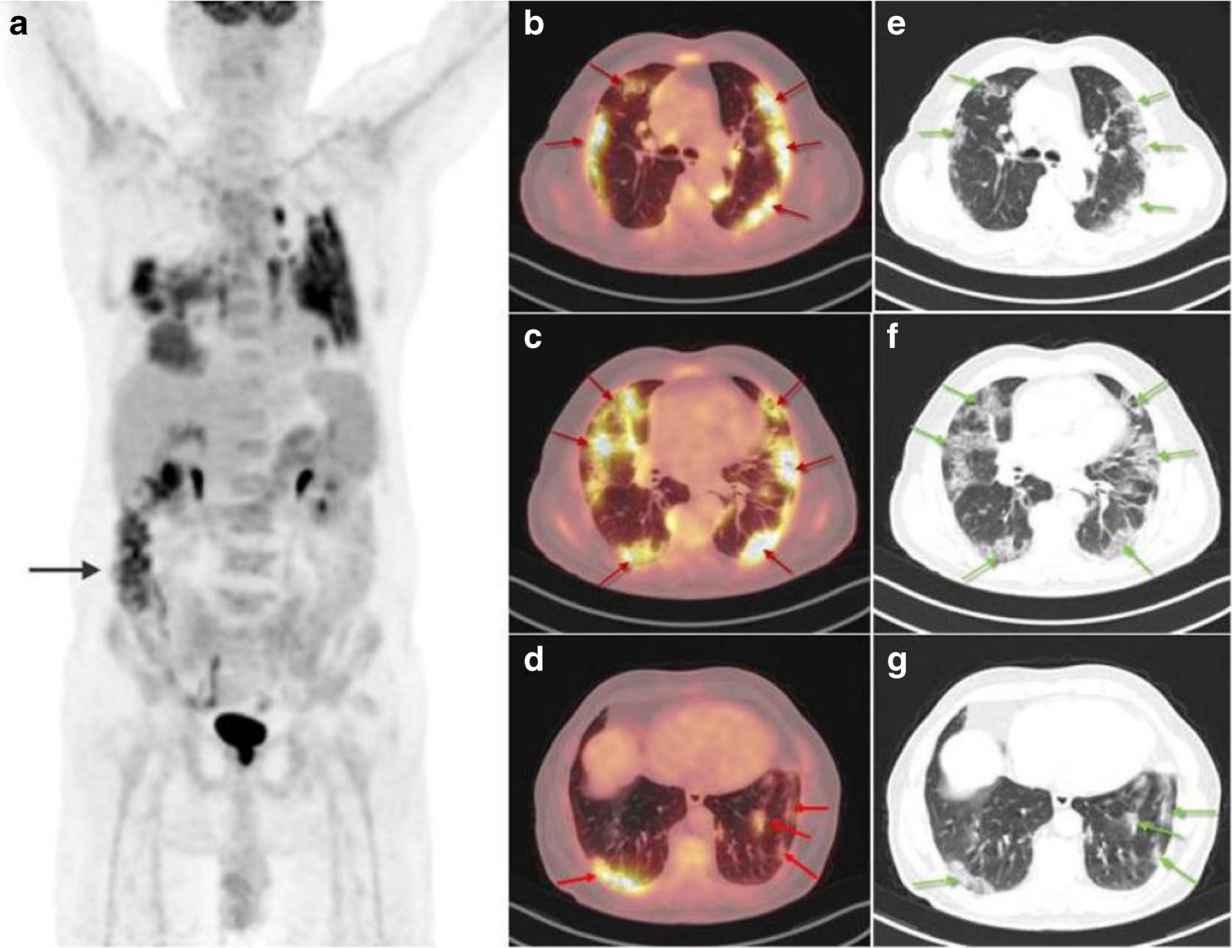
Incidental detection of COVID-19 in an 82-year-old patient with a history of adenocarcinoma of the colon. Increased ^18^F-FDG uptake (SUVmax 8.6) was observed in the bilateral lung fields (**a**, maximal intensity projection image; **b**–**d**, axial fused PET/CT images), corresponding to the bilateral ground-glass opacities on CT (**e**–**g**, axial CT images). Physiologic activity was also observed in the bowel indicated by a black arrow in the PET image (reproduced by permission from reference [27])

^18^F-Labeled fluorodeoxyglucose (^18^F-FDG), a radiolabeled glucose analogue, is still the most commonly-used PET imaging agent in COVID-19 cases, and the integrated PET/CT system can be utilized to characterize the functional and structural changes of COVID-19 simultaneously. COVID-19 pneumonia commonly manifested as ^18^F-FDG-avid foci in the lung on PET imaging (Fig. 2) and showed bilateral distribution of ground-glass opacities with or without consolidation on CT imaging (Fig. 2) [28]. The increased ^18^F-FDG uptake for pulmonary lesions of COVID-19 was related to the higher glycolytic activity of the recruited cells in the response to inflammation, especially the activated neutrophils, granulocytes, and macrophages [29, 30]. By using ^18^F-FDG PET/CT, early pathophysiological changes can be detected in the affected tissues with infectious or inflammatory diseases. Although there is no human clinical data to date on the onset time of pulmonary lesions after SARS-CoV-2 infection, preclinical studies have been conducted to explore the dynamic processes of COVID-19 abnormalities [31]. In macaques treated with bilateral intrabronchial instillation of SARS-CoV-2, lung imaging abnormalities such as ground-glass opacities, paving, and alveolar consolidation were detected by CT, and increased glucose metabolism was detected by PET in the CT-defined lung lesions at 2 days after virus exposure [31]. Besides, as a sensitive whole-body imaging modality, PET has played an important role in identifying asymptomatic COVID-19 patients. According to an Italian cohort study, which included 65 patients for standard oncologic indications within an 8-day period, interstitial pneumonia and regional increased ^18^F-FDG uptake were found in nearly 10% (6) of patients, and these patients were confirmed the diagnosis of COVID-19 or quarantined subsequently [32]. Another study including 1298 patients also showed a significant increase of interstitial lung alterations at ^18^F-FDG PET/CT from December 2019 to May 2020 (4.2%) than the control period in the previous year (1.9%) [33]. The difference became more pronounced in the period from January to May 2020 and reached the highest significance in March 2020 (7/83 patients, 8.4% vs. 3/134 patients, 2.2%, *p* = 0.001) [33]. The rate of interstitial pneumonia suspicious for COVID-19 detected by ^1^^8^F-FDG PET/CT could be even higher in a high-prevalence country [34]. Considering that the asymptomatic COVID-19 patients may also undergo a sudden worsening of clinical conditions and result in virus transmission, ^18^F-FDG PET has contributed greatly to identifying these patients that need isolation and treatment during the pandemic era [35, 36].

It should be noted that pulmonary manifestations of COVID-19 in PET/CT are not specific. Increased ^18^F-FDG uptake in the area of ground-glass opacities, the most remarkable imaging feature reported, could also be observed in a variety of diseases, especially for other pulmonary infections [37, 38]. To identify specific ^18^F-FDG PET imaging features of COVID-19, a recent study included 11 COVID+ patients and 11 COVID- subjects (8 bacterial pneumonia, 2 granulomatosis, and 1 lymphoma) and compared the differences between groups in imaging [39]. While there were no significant differences in CT abnormalities between these two groups, the SUVmax of lung lesions was significantly lower in the COVID+ group compared with that in the COVID− group (3.7 ± 1.9 vs. 6.9 ± 4.1, *p* = 0.03). As the COVID− group presented a trend of a smaller percentage of pulmonary involvement (6.7 ± 7.2 vs. 11.5 ± 8.0, *p* = 0.16) and higher rates of consolidation (64% vs. 27%, *p* = 0.09), the higher ^18^F-FDG SUVmax could be explained by the less non-cellular component in the ground-glass opacities [39]. This study may provide potential imaging information for the comparison between COVID-19 and bacterial pneumonia. Infections of the lower respiratory tract caused by other influenza viruses could also present similar pulmonary manifestations, such as H1N1, parainfluenza virus, adenovirus, and respiratory syncytial virus. However, limited data have been reported to demonstrate the differences of PET features between COVID-19 and other viral pneumonia. Subtle radiological features, such as pleural effusion, bronchiectasis, and bronchial wall-thickening, may help the differential diagnosis [37]. It was suggested that COVID-19 was more likely to have a peripheral distribution (80% vs. 57%), ground-glass opacity (91% vs. 68%), fine reticular opacity (56% vs. 22%), and vascular thickening (59% vs. 22%), but less likely to have a central + peripheral distribution (14% vs. 35%) and pleural effusion (4% vs. 39%) [40]. In addition, differential diagnosis with pulmonary malignancies and drug-induced pneumonia are also highly necessary, as PET is mainly applied for whole-body evaluation in oncological patients now [37, 41].

**Fig. 3:**
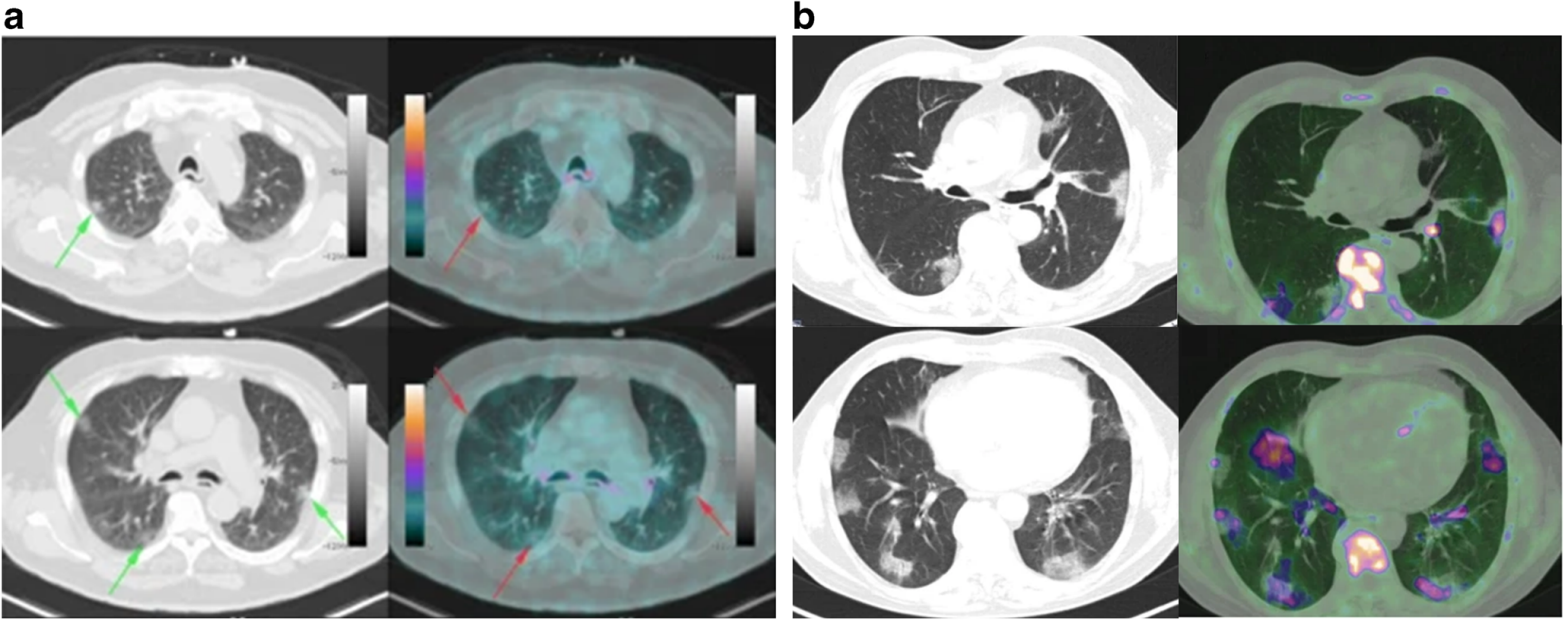
Examples of PET/CT images of COVID-19 patients using ^68^Ga-PSMA-11 and ^18^F-choline. **a** ^68^Ga-PSMA-11 PET/CT of an asymptomatic 66-year-old man, who requested for primary staging of prostate cancer. Peripheral ground-glass opacities on both lungs were observed on CT, with mild bronchial uptake of ^68^Ga-PSMA-11 (SUVmax 4.4). **b** ^18^F-choline PET/CT images of an asymptomatic 59-year-old man with biochemical recurrence of prostate cancer. Bilateral ground glass opacities were identified, with increased ^18^F-choline uptake (SUVmax range 3–4) (reproduced by permission from reference [42, 43])

Pulmonary lesions in COVID-19 patients could also be detected by PET using other radiotracers (Fig. 3). In prostate cancer patients with asymptomatic COVID-19, ^68^Ga-prostate-specific membrane antigen (PSMA) PET/CT was used for staging, and the results showed increased metabolism (SUVmax 2.5–3.2) in the ground-glass opacities in the lung [42, 44]. Though PET with ^68^Ga-PSMA was originally utilized to evaluate biochemical recurrence of prostate cancer, increased uptake of PSMA ligand has been reported in a variety of non-prostatic diseases, including inflammatory processes, bone lesions, benign tumors, and malignant neoplasms [45]. The increased uptake of ^68^Ga-PSMA in COVID-19 was speculated to result from infection-induced increased blood flow and upregulation of folate receptor expression in activated macrophages in the inflammation lesion [42, 44, 45]. By using PET/CT with ^18^F-Choline, a substrate for the synthesis of phosphatidylcholine that constitutes the essential component of the cell membrane, one patient with prostate cancer and asymptomatic COVID-19 showed mild choline uptake (SUVmax 3.8) in ground-glass and consolidative opacities in the lung, which may indicate the infiltration of activated macrophages with highly expressed choline kinase into the lung [43, 44]. These studies also demonstrated the necessity of further research to explore the role of PET, by using radiolabeled choline analogues, in imaging and quantifying the degree of lung inflammation, as well as stratifying the gravity of this disease [46]. Besides, considering the high expression of folate receptors in macrophages, PET imaging with folic acid analogue ^18^F-AzaFol could also be used to detect and monitor the activated macrophages in patients infected with COVID-19 [47]. Although PET data of COVID-19 using these radiopharmaceuticals is still limited up to now, those available radiotracers may be valuable to provide insight of inflammatory cells and pathophysiological changes in COVID-19 from specific perspectives.

Taken together, with increasing studies reporting the incidental detection of COVID-19 patients by using PET imaging, nuclear medicine physicians ought to be familiar with the pathophysiology and imaging manifestations of COVID-19, and remain vigilant to avoid missed diagnosis or misdiagnosis of asymptomatic COVID-19 in the pandemic era.

## PET in the prognosis evaluation of COVID-19

Accompanied by the release of chemokines and the infiltration of inflammatory cells caused by viral infection, the glucose uptake of the infected foci would significantly increase [29]. The increased uptake of ^18^F-FDG thus has the potential to indicate the location and degree of inflammation. Recent studies have also suggested that PET may play a role in evaluating the prognosis of COVID-19 [48–50].

The values of SUVmax in incidentally detected COVID-19 infections usually range from 2.2 to 18 [51]. According to some case observations, it was suggested that a higher SUVmax might indicate a longer recovery time for the COVID-19 patients [48], as one subject with an SUVmax of 4.6 recovered at approximately 17 days after the symptom occurrence, while another patient had a SUVmax of 12.2 did not recover until 26 days since the onset of symptoms [49]. Similarly, in another case with an SUVmax of 4.9, a 15-day recovery time was observed [50]. Certainly, caution is required when applying such a principle in clinical practice, as contradictory results have also been reported on the predictive value of SUVmax. In a prospective study, 13 patients were included and classified as “inflammatory” and “low inflammatory” according to the hypermetabolic volume and SUVmax, then the short-term clinical outcome of those patients was adjudicated based on clinical and biological data during 3 days before and after the PET scan. No correlation was observed between PET pulmonary inflammatory status and short-term clinical outcome [52]. However, this study also suffered from limited statistical power, especially that no significant relationship between biological markers of inflammation and pulmonary ^18^F-FDG metabolism was observed [52]. Since the lack of large-sample data, it would be of interest to explore the relation between lesion SUV and patient prognosis, which would be helpful to identify the patient with a poor prognosis that requires great concern.

**Fig. 4:**
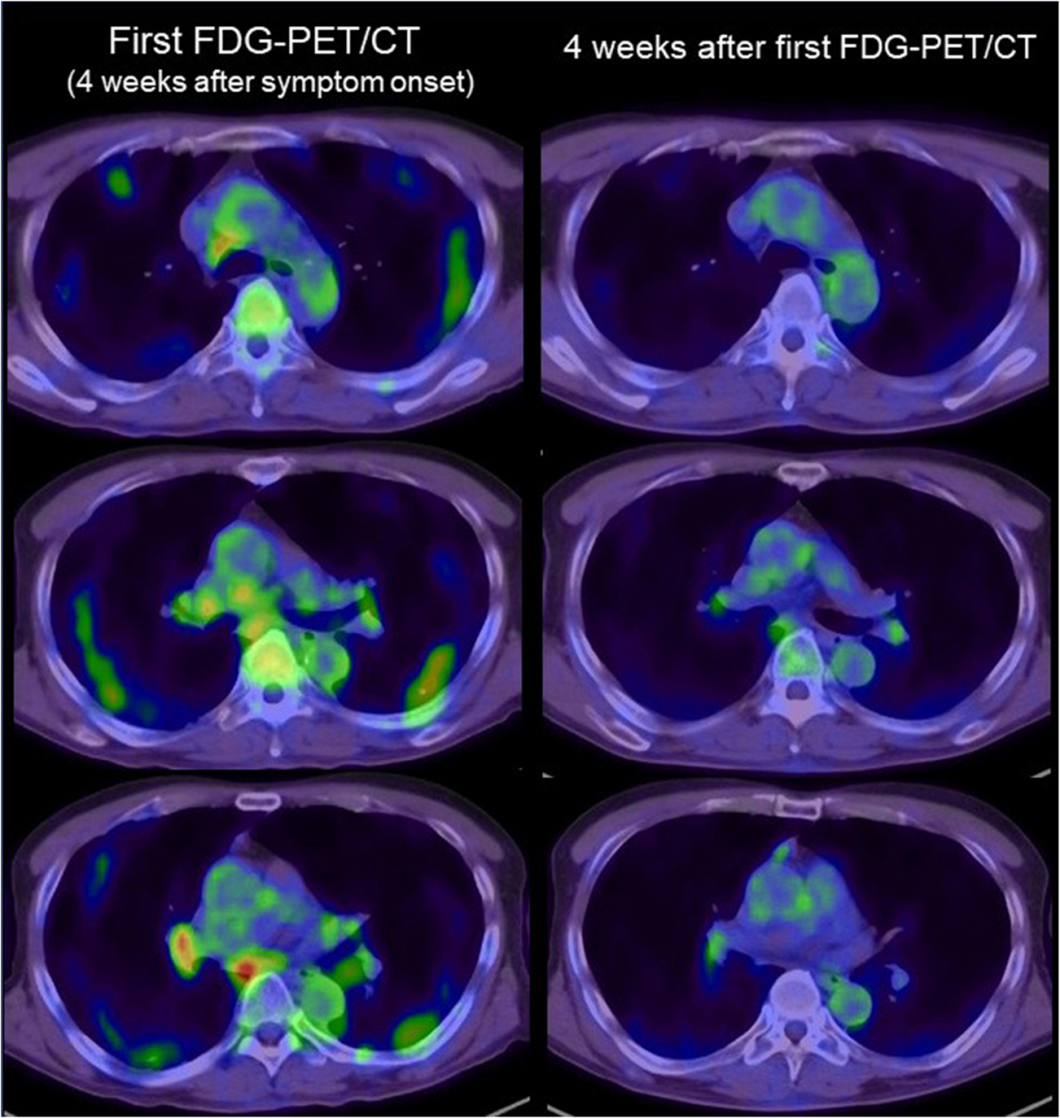
^18^F-FDG PET/CT images of lymph nodes in a COVID-19 patient. Increased ^18^F-FDG uptake was observed in mediastinal and hilar lymph nodes in the first scan 4 weeks after the symptom occurrence. The ^18^F-FDG uptake was decreased in the second examination 4 weeks after first scan (reproduced by permission from reference [53])

Though not specific to COVID-19, the presence of lymph node abnormality in PET imaging also provides a potential value for predicting the infection severity of COVID-19, which is comparable to many other infections. For COVID-19, hypermetabolic lymph nodes were commonly reported in the hilum, supraclavicular, and mediastinal area (Fig. 4) [49, 52, 53]. Compared with conventional imaging modalities, PET possessed higher sensitivity in detecting lymph node involvement [54, 55]. Indeed, even for some lymph nodes that were not anatomically enlarged, increased uptake of ^18^F-FDG could be observed [49]. Moreover, the increased uptake of ^18^F-FDG in lymph nodes can be observed as early as a slightly increased number of monocytes in the circulation system (within the normal range) occurred in rhesus macaques infected by MERS-CoV, with a significant correlation between the influx rate constant of ^18^F-FDG in the lymph nodes and the circulating level of monocytes [56]. In clinical studies of COVID-19, lymph node enlargement has been considered an indicator of the infection severity and was used to indicate a worse prognosis [57, 58]. In another case report, researchers have proposed that higher SUVmax values of lymph nodes could herald a more severe condition of the SARS-CoV-2 infection, which further underscore the role of lymphadenopathy as a predictor of a worse outcome [59]. In addition, some studies reported that the lesions of mediastinal lymph nodes with high metabolism did not spatially match the distribution of the pulmonary inflammation, suggesting that the glucose uptake in lymph node might be an independent imaging marker of COVID-19 [59, 60]. However, considering the limited number of studies as well as the small sample size, further studies are essential to explore the potential role of hypermetabolism in lymph nodes detected by PET in the management of COVID-19.

It is worth noting that the hypermetabolism on ^18^F-FDG PET could also be observed in COVID-19 recovered patients. For example, in a COVID-19-recovered patient who showed negative nucleic acid test results and reduced ground-glass opacity in CT 2 months post-discharge, ^18^F-FDG-positive lesion remained both in the lungs and lymph nodes [61]. Besides, the inflammatory axilla lymph nodes in PET imaging have been reported in people administered the COVID-19 vaccine [62]. As the global vaccination effort continues, knowledge of the potential for COVID-19 vaccine-related ipsilateral adenopathy is necessary [63]. These studies further emphasized the necessity of an integrated consideration when interpreting PET images.

## Potential role of PET in COVID-19-derived complications

### PET in pulmonary complications

Although most COVID-19 patients presented only mild to moderate symptoms, SARS-CoV-2 can cause severe acute pulmonary complications (including ARDS, pulmonary embolism, etc.) and even death, especially among the elderly or patients with preexisting chronic diseases.

ARDS is one of the most serious pulmonary complications of COVID-19 and usually occurs 10 days after the onset of symptoms [64, 65]. According to a global literature survey that included 2486 hospitalized COVID-19 patients, the incidence and mortality rates were approximately 33% and 16%, respectively [66]. In severe cases transferred to an ICU, the incidence of ARDS was even up to 75% [66]. As there is no specific treatment for this complication currently, early recognition is crucial for subsequent therapy [67]. It has been reported that at the early stage of ARDS, diffuse lung uptake of ^18^F-FDG was detected by PET, while no obvious changes were observed in CT images [68]. In another study, researchers investigated eight chest trauma and lung contusion cases to evaluate the role of ^18^F-FDG uptake in predicting the occurrence of ARDS [69]. In this study, four patients developed ARDS within 1–3 days following PET examination, and three of them showed diffuse ^18^F-FDG uptake, while those without ARDS showed significant ^18^F-FDG uptake only in the focal pulmonary opacity area, suggesting the predictive value of ^18^F-FDG PET in the occurrence of ARDS [69, 70]. As the successful management of ARDS requires rapid adjustments for therapeutic regimen, ^18^F-FDG PET/CT may help in predicting the progression of COVID-19 into ARDS and provide an attractive method for therapeutic assessment. Besides, considering the important role of macrophages in the occurrence and progression of COVID-19, some other radiotracers, such as ^18^F-AzaFol and ^18^F-Choline, may also have the potential to screen suitable patients and monitor the treatment response in anti-macrophage therapies [43, 71, 72].

Pulmonary embolism is also an important complication for patients with COVID-19. COVID-19 induced the hypercoagulable state, which may lead to deep vein thrombosis and subsequent pulmonary embolism events [73]. According to a meta-analysis including 1835 COVID-19 patients, the incidence and mortality rate of COVID-19 patients who developed pulmonary embolism was 15.3% and 45.1%, respectively [74], significantly higher than the incidence rate detected in seasonal and pandemic influenza (~3%) [75], and the mortality rate for general in-hospital cases (~4%) [76]. CT pulmonary angiography is the gold standard of pulmonary embolism, with MR angiography as an alternative [77]. It is noteworthy that lung scintigraphy remains the best nuclear medicine technique to study pulmonary embolism, and its value on COVID-19 patients has been discussed [78]. PET studies on COVID-19-related pulmonary embolism are still rarely reported. Simultaneous occurrence of COVID-19 pneumonia and pulmonary embolism in the ^18^F-FDG PET images were reported in a case report of one tumor patient, however, no meaningful imaging biomarker was observed [79]. With reference to related mechanisms and experience, PET may have a greater role in the detection of venous thromboembolism compared with the detection of pulmonary embolism [80, 81]. A prospective study evaluated the significance of ^18^F-FDG PET in the detection of lower extremity venous thrombosis and pulmonary embolism [82]. The results showed that physicians diagnosed all 7 patients with lower extremity venous thrombosis based on the hypermetabolism of thrombus and blood vessels, but only 2 of 6 patients with pulmonary embolism were accurately diagnosed [82]. Therefore, when interpreting the images of suspected patients with COVID-19, it is essential to pay attention to the manifestation of vascular inflammation and thrombosis, so as to better prevent COVID-19-related pulmonary embolism.

**Fig. 5:**
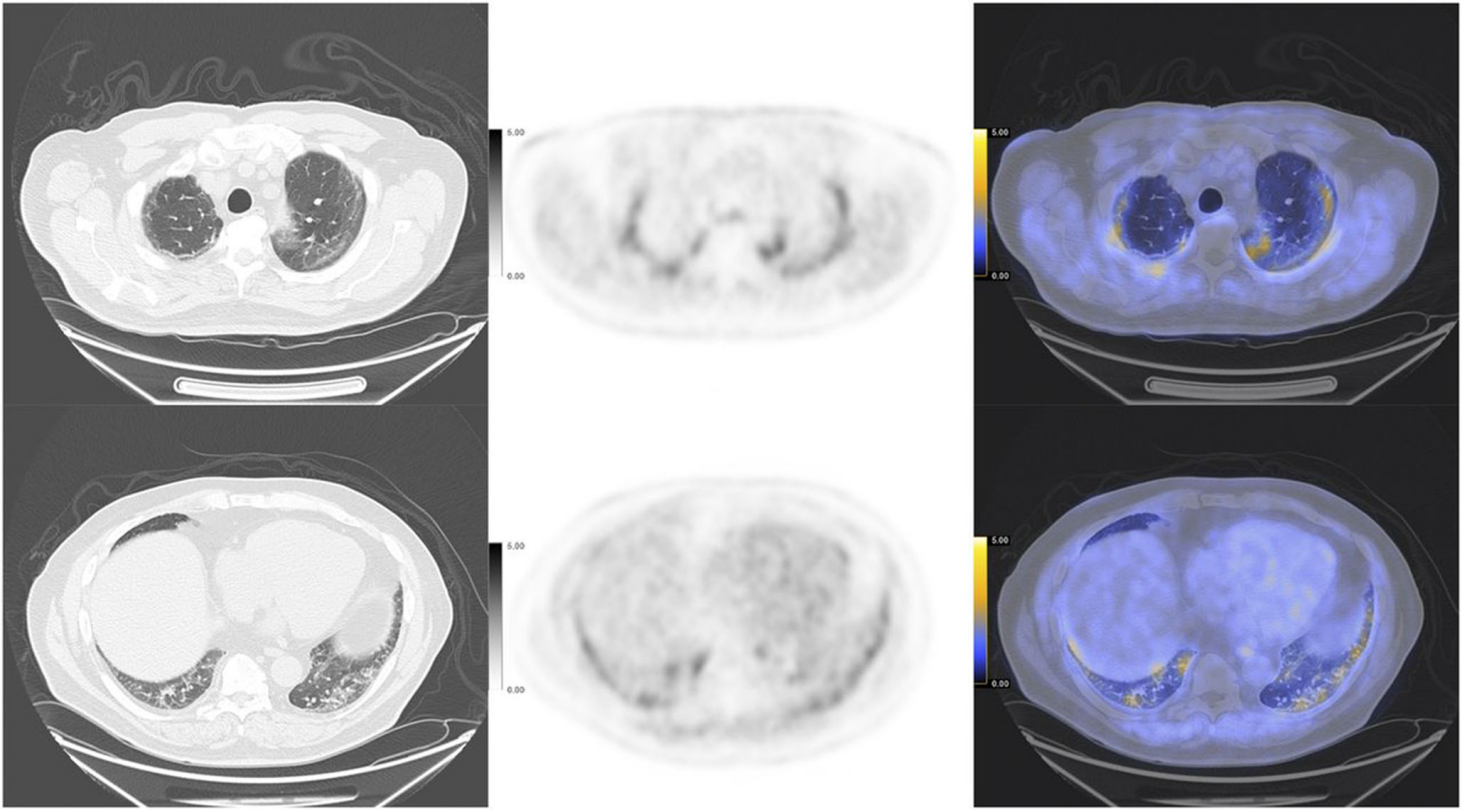
^18^F-αvβ6-BP PET/CT images of a COVID-19-recovered patient 2 months after infection. Axial CT (left), ^18^F-αvβ6-BP PET (middle), and fused PET/CT (right) images showed increased ^18^F-αvβ6-BP uptake (SUVmax 5) and bilateral patchy opacity (reproduced by permission from reference [91])

Although the long-term effect of COVID-19 on the lung remains unclear, some researchers have called for attention to the pulmonary fibrosis in COVID-19-recovered patients [83]. Actually, patients with severe COVID-19 and those with idiopathic pulmonary fibrosis shared similar prevalence factors, including aging, male, comorbidities with hypertension, diabetes, etc. [84], and a considerable number of patients with ARDS complication would experience long-term lung function impairment and pulmonary fibrosis [85, 86]. Considering the anatomical alternations detected by CT are caused by major changes of the tissue, molecular imaging method is highly valuable for visualizing the process of pulmonary fibrosis in the diagnosis and treatment. PET can detect dynamic changes of key proteins in the pathophysiological process of pulmonary fibrosis, for example, type I collagen, of which the deposition is a hallmark of idiopathic pulmonary fibrosis [87], and αvβ6, a kind of arginine-glycine-aspartic acid (RGD)-integrin involved in the pathogenesis of pulmonary fibrosis by activating transforming growth factor β (TGFβ) [88]. By using PET with ^68^Ga-CBP8, which binds to type I collagen, PET imaging showed increased collagen tracer uptake in fibrotic lung regions determined by chest CT and the normal regions on CT [89]. Through PET with ^18^F-FB-A20FMDV2, an αvβ6-selective ligand, researchers found that the αvβ6 protein was significantly upregulated in patients with pulmonary fibrosis compared with normal people [90]. And by PET using integrin αvβ6-binding peptide, ^18^F-αvβ6-BP, in a COVID-19-recovered patient 2 months after infection, an increase in the uptake of the tracer in the opaque area was observed, while the uptake in normal lung parenchyma remained low (Fig. 5) [91]. This study has provided preliminary but crucial evidence for the role of PET in monitoring the persistence/progress of pulmonary fibrosis in patients recovered from COVID-19 [91]. Based on the existing data, long-term follow-up for patients recovered from COVID-19 is strongly recommended. Further studies should determine the incidence as well as severity of pulmonary fibrosis in this population and pay special attention on the prognostic value of molecular imaging in the progression and treatment of pulmonary fibrosis.

### PET in neurological complications

Although pulmonary manifestations constitute main presentations of the COVID-19, neurological abnormalities have roused increasing concern of doctors and scientists [92]. More than one-third of patients had neurologic manifestations, including central nervous system manifestations (e.g., dizziness, headache, consciousness impairment, acute cerebrovascular disease, ataxia, and seizure), peripheral nervous system manifestations (e.g., taste impairment, olfactory impairment, vision impairment, and nerve pain), and manifestations of skeletal muscular injury [93, 94]. In another meta-analysis, results indicated 65% COVID-19 patients got delirium, 69% for agitation and 21% for altered consciousness in the post-illness stage [95].

**Fig. 6:**
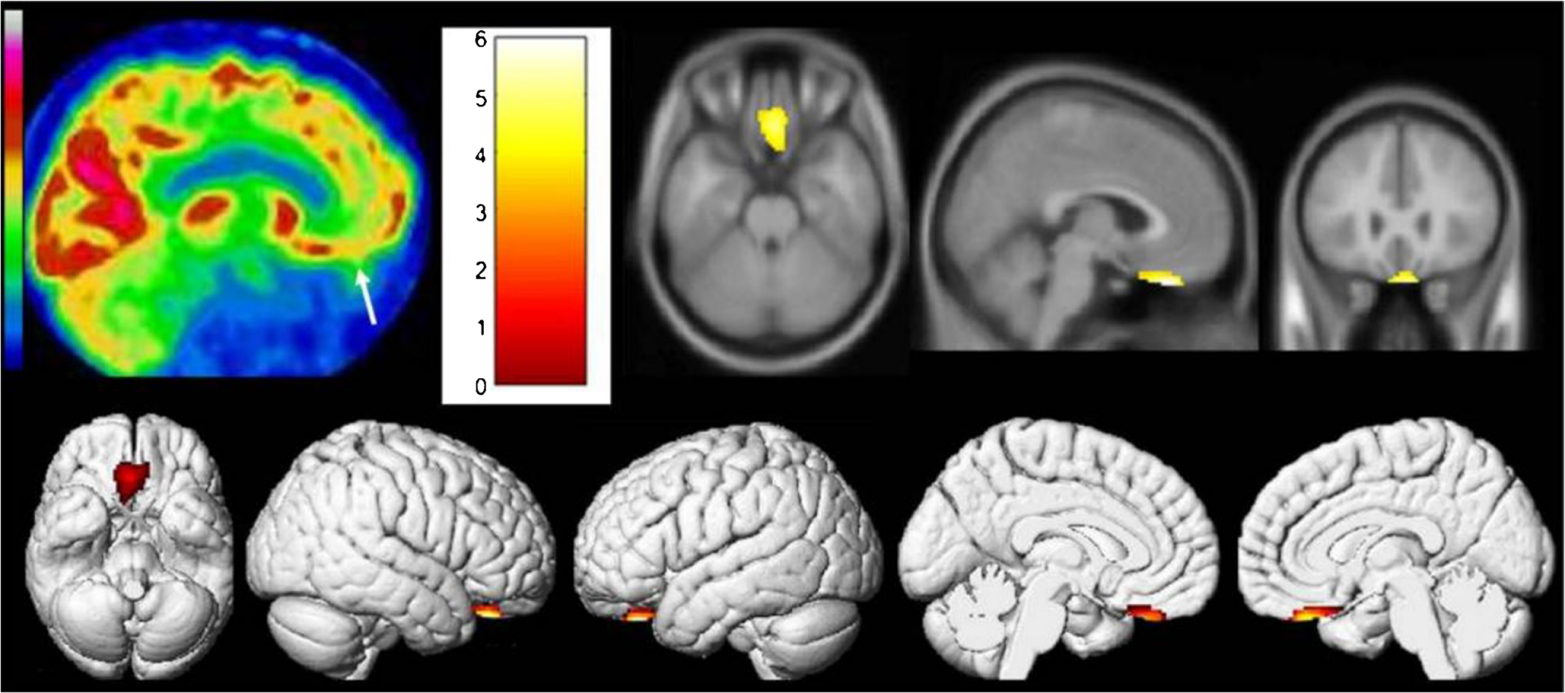
Brain ^18^F-FDG PET image of a COVID-19 patient with 4-week prolonged anosmia. Hypometabolism was observed in the olfactory/rectal gyrus (white arrow) and confirmed by voxel-to-voxel comparison with healthy subjects in SPM analysis (p voxel < 0.001, p-cluster < 0.05, uncorrected) (reproduced by permission from reference [97])

PET plays a unique role in exploring SARS-CoV-2 infection mechanisms and evaluating the neural invasion, despite the cancels or delays of non-urgent nuclear testing in the COVID-19 pandemic. As neurotropic and neuroinvasive capabilities of other coronaviruses including HCoV-OC43, MERS-CoV, and SARS-CoV described before, coronavirus infected deeper parts of the brain across the cribriform plate of the ethmoid bone, from the nose to the olfactory epithelium, and finally lead to neurological disorders such as multiple sclerosis and encephalomyelitis [96, 97]. Given the anatomical proximity of neurons, nerve fibers, and mucosa within the oro- and nasopharynx, as well as taste and smell impairment observed in COVID-19 patients, SARS-CoV-2 is presumed to invade the nervous system in a similar way. ^18^F-FDG PET showed the hypometabolism of the olfactory/rectus gyrus in one patient with 4-week prolonged anosmia (Fig. 6), and hypometabolisms within the amygdala, hippocampus, parahippocampus, cingulate cortex, pre−/post-central gyrus, thalamus/hypothalamus, cerebellum, pons, and medulla in another patient who complained of delayed onset of a painful syndrome. The variance of the ^18^F-FDG metabolic pattern may indicate a different distribution of affected brain regions. Therefore, researchers hypothesized that SARS-CoV-2 possibly got access to the brain through the olfactory bulb and caused extensive damage to other brain structures. This hypothesis was proved soon in later researches [96, 97]. The evidence that CoV particles together with SARS-CoV-2 RNA existed in the olfactory mucosa, as well as in neuroanatomical areas receiving olfactory tract projection, indicated that SARS-CoV-2 protruded the nervous system by crossing the neural–mucosal interface in the olfactory mucosa, according to autopsy material from individuals with COVID-19 [98].

In addition to directly viral neuroinvasion, SARS-CoV-2 may also cause indirect neural impairment through immune inflammation. Researchers have reported four cases of COVID-19-related encephalopathy with varied clinical presentations, as well as negative SARS-CoV-2 RT-PCR in the cerebrospinal fluid (CSF). However, all of them had consistent brain ^18^F-FDG PET pattern of abnormalities and without MRI features of encephalitis nor significant CSF abnormalities. Most importantly, they all recovered after immunotherapy [99]. All the pieces of evidence suggested that host immunity was another way to mediate damage of the central nervous system through cytokine storm and antibody, which had been proved in latter autopsy studies. Through studying tissue specimens of COVID-19 patients, researchers found that early activated macrophages responding to SARS-CoV-2 infection initiated and regulated an immune cascade, which was the direct evidence of SARS-CoV-2-mediated neuroinflammatory response [98]. In addition, PET pattern of abnormalities appeared earlier than MRI and nucleic acid test results, which meant PET was a promising noninvasive approach to avoid delayed diagnosis and treatment and prevent further transmission. A case with concomitant autoimmune encephalitis and SARS-CoV-2 infection also confirmed this view. The patient presented diffuse cortical hypometabolism associated with putaminal and cerebellar hypermetabolism in ^18^F-FDG PET and normal brain in MRI. The inconsistence between ^18^F-FDG PET and MRI emphasized the potential of metabolism in PET as an early biomarker for cerebral abnormality in COVID-19 [100].

The acute brain damage caused by neuroinvasion of SARS-CoV-2 complicates the diagnosis and treatment for COVID-19 infection. And the SARS-CoV-2-mediated neuroinflammatory response also increases the risk of neuropsychiatric and neurodegenerative disorders in the recovered patients. Thus, it is essential to pay much greater attention to acute brain damage and chronic neurological sequelae. What’s more, social isolation, economic recession, and social boycott trigged by the COVID-19 pandemic also induced unimaginable mental sufferings such as depression, panic, fear, anxiety, stress, trauma, and adjustment disorder [101, 102]. All the factors make the differential diagnosis of neuropsychiatric and neurodegenerative disorders more complicated in the COVID-19 epidemic [103]. PET has unique advantages in early detection of neurological abnormalities, differential diagnosis of the main neurological disorders, and long-term tracking of neurological sequelae [104]. However, current PET data of COVID-19 patients is rare and unsystematic because of less use of ^18^F-FDG PET in an emergency setting or infectious disease [49]. Besides, mid-term and long-term consequences of SARS-CoV-2 neuro-infection are not completely clear considering only 1 year since the first public reported case was reported. Therefore, it is necessary to conduct post-viral cohort studies with longer follow-up to specify the exact dynamic changes of metabolism or neurotransmitters on PET longitudinally, so as to explore the mechanisms of long-term neurological complications and potential prevention methods.

### PET in other extrapulmonary complications

SARS-CoV-2 infection also causes acute myocardial injury and chronic damage in the cardiovascular system, especially in patients with underlying cardiovascular diseases [105]. More than a quarter of hospitalized COVID-19 patients had myocardial injury, some of whom with serious cardiac complications, including biventricular heart failure, arrhythmias, and occasionally cardiogenic shock [106, 107]. And according to a prospective observational cohort study, ongoing myocardial inflammation could exist in a significant proportion (60 of 100) of recovered patients [108]. Although cardiac nuclear imaging has minimal utility in managing the acute stages of COVID-19 patients, PET has unique value in the cardiac function assessment for those recovered from COVID-19, including evaluating myocardial ischemia, chest pain syndromes, myocardial viability, amyloidosis, and infections in implanted devices [109]. Besides, PET also serves as a useful complement to anatomic imaging modalities such as cardiovascular magnetic resonance and cardiac ultrasound in patients with complex situation, such as stents, significant coronary calcification, dye allergy, and risk of worsening renal function [109].

In ten patients recovered from COVID-19 complaining persistent symptoms, such as dyspnea, fatigue, joint pain, chest pain, and trembling hands, visual analysis revealed increased target-to-blood pool ratio in some vascular regions in ^18^F-FDG PET images, suggesting the vascular inflammation might be potential causes of the persisting symptoms [110]. In another observational study, which included 160 patients during the initial 3 weeks of UK lockdown, extrapulmonary findings were reported in 7% of the case group, including the increased ^18^F-FDG uptake of tonsillar, salivary gland, and gastrointestinal tract [111]. These studies further underlined the importance of evaluating extrathoracic findings in COVID-19 patients by using PET imaging.

## PET in developing novel therapies for COVID-19

Though there is no specific treatment for COVID-19 currently, a variety of novel therapies are being developed. PET molecular imaging is of great potential to accelerate the development processes, especially in the translation of experimental therapies into the clinic, and the validation of therapeutic efficiency.

By using novel screening strategies such as structure-based drug design, virtual screening, and high-throughput screening methods, the discovery processes of drug leads that target SARS-CoV-2 and SARS-CoV-2-infected cells have been accelerated [112]. And it is crucial to identify the most promising drug candidates and terminate those not suitable for clinical use in order to achieve a rapid and effective drug discovery [113]. As molecular imaging enables the visualization and quantification of radiolabeled drugs, PET is of great potential to help the antiviral drug development [114]. For example, PET imaging with the radiolabeled drug candidates in living subjects could facilitate to characterize the pharmacokinetic properties, visualize the distribution of drugs within and outside the target area, and explore the potential intermolecular interactions. Moreover, once an appropriate compound is obtained, this radiolabeled drug candidate, in turn, can also be used to assess the location and degree of SARS-CoV-2 infection, with a high specificity [115].

Theranostic approaches based on radiolabeled antibodies and drugs could also be a way worth a try. In such a therapeutic strategy, radionuclides emitting radiation could be used either for imaging or treatment. The radiation would not only kill the virus-infected cell directly but also trigger immunomodulation reactions such as the production of natural killer cells and interferon to achieve the anti-virus effects [116]. Currently, a variety of radionuclides have been used as theranostic pairs in various malignancies, including ^68^Ga-^177^Lu, ^68^Ga-^90^Y, ^64^Cu-^67^Cu, ^83^Sr-^89^Sr, and ^86^Y-^90^Y [117, 118]. And the antiviral drugs or antibodies that tested for SARS-CoV and MERS-CoV could be potential carriers to target the virus. Especially, as the SARS-CoV and SARS-CoV-2 shared the functional host receptor, angiotensin-converting enzyme 2 (ACE2), antiviral drugs/antibodies for SARS could be feasible candidates in the developing of radiolabeled theranostic treatment for COVID-19 [119].

In addition to targeting the virus or infected cells, modulating macrophage activation may be another promising approach for the treatment and detection of COVID-19 [120]. Monocytes/macrophages are the principal leukocytes that accumulated in the initial phase of the host response to the SARS-CoV-2 infection and serve as the first antiviral defense through the production of interferon. Besides, the virus could be spread across different organs with the migration of virus-infected monocytes and macrophages [121]. The use of monocyte/macrophage (GM-CSF) inhibitors, which inhibited the differentiation and migration of monocyte/macrophage, was proposed as a potential therapeutic approach for COVID-19 [122]. Thus, the imaging of macrophages may help to visualize both the host immune response and the virus migration. As mentioned above, COVID-19 lesions showed increased choline metabolism in ^18^F-Choline imaging [43, 44]. And numerous novel macrophage-target radiotracers, such as ^18^F-PBR06, ^64^Cu-DOTATATE, ^18^F-PEG-FOLATE, and ^68^Ga-pentixafor, have been established with high specificity [123]. These radiotracers could be valuable in the development of macrophage-modulating therapies.

## Conclusions and future perspectives

During the past one year, increasing cases and research of PET on COVID-19 have been reported. Though not recommended as a routine examination for COVID-19 in clinical practice, PET has played an important role in detecting asymptomatic COVID-19 patients, especially in the oncological whole-body evaluation. These results helped to reveal functional changes of COVID-19 in molecular perspectives. Further studies with a large sample size are of interest to better determine the PET imaging features of COVID-19 and explore the potential predictive imaging biomarker of clinical outcome. Besides, given the huge number of cumulative confirmed COVID-19 patients currently, potential COVID-19 complications may lead to another global health burden. PET is of great potential in the identification and staging of the patients with COVID-19 complications, as well as the exploration of underlying mechanisms. Further research should determine the incidence and severity of COVID-19 complications, especially the potential long-term complications, and developing imaging methods to visualize and quantify the pathophysiological changes of COVID-19 complications. Additionally, novel drugs and therapeutic strategies for COVID-19 remain challenging currently. In the development of novel therapies for COVID-19, it is of great importance to take full advantage of PET and accelerate the developing processes.

Overall, PET is of great potential in the improvement of management for COVID-19 patients, but further in-depth research is requisite. As more PET data on COVID-19 are accumulated, reliable imaging-based COVID-19 identification could be achieved, and the imaging features could be used to aid the management of pneumonia as well as concurrent complications. By using multiple specific radiotracers, the interaction between the SARS-CoV-2 and the body could be visualized, facilitating the development of novel therapeutic strategies. Besides, researchers in the field of molecular imaging would be deeply involved in the development of COVID-19-targeted drugs, contributing in the ex vivo screening and in vivo validation. With the role of PET in the COVID-19 pandemic era be adequately explored, the paradigm could also be applied in combating various other epidemics in the future.

## References

[1] Zhu N, Zhang D, Wang W, Li X, Yang B, Song J (2020). A novel coronavirus from patients with pneumonia in China, 2019. N Engl J Med.

[2] Salzberger B, Glück T, Ehrenstein B. Successful containment of COVID-19: the WHO-report on the COVID-19 outbreak in China: Springer; 2020. 10.1007/s15010-020-01409-4 PMC7095462 32185635

[3] Yan Y, Pan HS, Shao N, Xuan Y, Wang SF, Li WJ (2020). COVID-19 in Singapore: another story of success. International Journal of Mathematics for Industry.

[4] Leung TYM, Chan AYL, Chan EW, Chan VKY, Chui CSL, Cowling BJ (2020). Short- and potential long-term adverse health outcomes of COVID-19: a rapid review. Emerg Microbes Infect.

[5] Cirulli E, Barrett KMS, Riffle S, Bolze A, Neveux I, Dabe S, et al. Long-term COVID-19 symptoms in a large unselected population. medrxiv. 2020.

[6] Tian M, He X, Jin C, He X, Wu S, Zhou R, et al. Transpathology: molecular imaging-based pathology. Eur J Nucl Med Mol Imaging. 2021. 10.1007/s00259-021-05234-1. 10.1007/s00259-021-05234-1 PMC8241651 33585964

[7] Zhang K, Sun Y, Wu S, Zhou M, Zhang X, Zhou R, et al. Systematic imaging in medicine: a comprehensive review. Eur J Nucl Med Mol Imaging. 2020. 10.1007/s00259-020-05107-z. 10.1007/s00259-020-05107-z 33210241

[8] Calais J, Fendler WP, Eiber M, Gartmann J, Chu FI, Nickols NG (2018). Impact of (68)Ga-PSMA-11 PET/CT on the management of prostate cancer patients with biochemical recurrence. J Nucl Med.

[9] Finnema SJ, Nabulsi NB, Eid T, Detyniecki K, Lin SF, Chen MK (2016). Imaging synaptic density in the living human brain. Sci Transl Med.

[10] Gao Y, Wu S, Pan J, Zhang K, Li X, Xu Y, et al. CRISPR/Cas9-edited triple-fusion reporter gene imaging of dynamics and function of transplanted human urinary-induced pluripotent stem cell-derived cardiomyocytes. Eur J Nucl Med Mol Imaging. 2020:1–13. 10.1007/s00259-020-05087-0. 10.1007/s00259-020-05087-0 33216174

[11] World Health Organization. Novel Coronavirus(2019-nCoV), Situation report- 1. https://www.who.int/docs/default-source/coronaviruse/situation-reports/20200121-sitrep-1-2019-ncov.pdf?sfvrsn=20a99c10_4. Accessed Jan 2020.

[12] Gorbalenya AE, Baker SC, Baric R, Groot RJd, Drosten C, Gulyaeva AA et al. Severe acute respiratory syndrome-related coronavirus: the species and its viruses–a statement of the Coronavirus Study Group. 2020.

[13] Gupta A, Madhavan MV, Sehgal K, Nair N, Mahajan S, Sehrawat TS (2020). Extrapulmonary manifestations of COVID-19. Nat Med.

[14] Wiersinga WJ, Rhodes A, Cheng AC, Peacock SJ, Prescott HC (2020). Pathophysiology, transmission, diagnosis, and treatment of coronavirus disease 2019 (COVID-19): a review. JAMA..

[15] Ai T, Yang Z, Hou H, Zhan C, Chen C, Lv W (2020). Correlation of chest CT and RT-PCR testing for coronavirus disease 2019 (COVID-19) in China: a report of 1014 cases. Radiology..

[16] Siemieniuk RA, Bartoszko JJ, Ge L, Zeraatkar D, Izcovich A, Kum E (2020). Drug treatments for covid-19: living systematic review and network meta-analysis. BMJ..

[17] Hasan SS, Capstick T, Ahmed R, Kow CS, Mazhar F, Merchant HA (2020). Mortality in COVID-19 patients with acute respiratory distress syndrome and corticosteroids use: a systematic review and meta-analysis. Expert Rev Respir Med.

[18] Meyerowitz-Katz G, Merone L (2020). A systematic review and meta-analysis of published research data on COVID-19 infection fatality rates. Int J Infect Dis.

[19] Lazzerini M, Barbi E, Apicella A, Marchetti F, Cardinale F, Trobia G (2020). Delayed access or provision of care in Italy resulting from fear of COVID-19. Lancet Child Adolesc Health.

[20] Freudenberg LS, Paez D, Giammarile F, Cerci J, Modiselle M, Pascual TNB (2020). Global impact of COVID-19 on nuclear medicine departments: an international survey in April 2020. J Nucl Med.

[21] Annunziata S, Bauckneht M, Albano D, Argiroffi G, Calabro D, Abenavoli E (2020). Impact of the COVID-19 pandemic in nuclear medicine departments: preliminary report of the first international survey. Eur J Nucl Med Mol Imaging.

[22] Kissler SM, Tedijanto C, Goldstein E, Grad YH, Lipsitch M (2020). Projecting the transmission dynamics of SARS-CoV-2 through the postpandemic period. Science.

[23] Paez D, Gnanasegaran G, Fanti S, Bomanji J, Hacker M, Sathekge M (2020). COVID-19 pandemic: guidance for nuclear medicine departments. Eur J Nucl Med Mol Imaging.

[24] Huang H, Gnanasegaran G, Paez D, Fanti S, Hacker M, Sathekge M, et al. Nuclear medicine services after COVID-19: gearing up back to normality: Springer; 2020. 10.1007/s00259-020-04848-1 PMC7197920 32367256

[25] Treglia G. The role of (18)F-FDG PET for COVID-19 infection: myth versus reality. Clin Transl Imaging. 2020:1–2. 10.1007/s40336-020-00367-z. 10.1007/s40336-020-00367-z PMC7191553 32355659

[26] Annunziata S, Delgado Bolton RC, Kamani CH, Prior JO, Albano D, Bertagna F et al. Role of 2-[(18)F]FDG as a radiopharmaceutical for PET/CT in patients with COVID-19: a systematic review. Pharmaceuticals (Basel). 2020;13(11). 10.3390/ph13110377. 10.3390/ph13110377 PMC7696195 33182811

[27] Amini H, Divband G, Montahaei Z, Dehghani T, Kaviani H, Adinehpour Z (2020). A case of COVID-19 lung infection first detected by [18F]FDG PET-CT. Eur J Nucl Med Mol Imaging.

[28] Ye Z, Zhang Y, Wang Y, Huang Z, Song B (2020). Chest CT manifestations of new coronavirus disease 2019 (COVID-19): a pictorial review. Eur Radiol.

[29] Jones HA, Marino PS, Shakur BH, Morrell NW (2003). In vivo assessment of lung inflammatory cell activity in patients with COPD and asthma. Eur Respir J.

[30] Deng Y, Lei L, Chen Y, Zhang W (2020). The potential added value of FDG PET/CT for COVID-19 pneumonia. Eur J Nucl Med Mol Imaging.

[31] Finch CL, Crozier I, Lee JH, Byrum R, Cooper TK, Liang J, et al. Characteristic and quantifiable COVID-19-like abnormalities in CT- and PET/CT-imaged lungs of SARS-CoV-2-infected crab-eating macaques (*Macaca fascicularis*). bioRxiv. 2020. 10.1101/2020.05.14.096727.

[32] Albano D, Bertagna F, Bertoli M, Bosio G, Lucchini S, Motta F (2020). Incidental findings suggestive of COVID-19 in asymptomatic patients undergoing nuclear medicine procedures in a high-prevalence region. J Nucl Med.

[33] Setti L, Bonacina M, Meroni R, Kirienko M, Galli F, Dalto SC, et al. Increased incidence of interstitial pneumonia detected on [(18)F]-FDG-PET/CT in asymptomatic cancer patients during COVID-19 pandemic in Lombardy: a casualty or COVID-19 infection? Eur J Nucl Med Mol Imaging. 2020. 10.1007/s00259-020-05027-y. 10.1007/s00259-020-05027-y PMC7480211 32909090

[34] Albano D, Bertagna F, Alongi P, Baldari S, Baldoncini A, Bartolomei M, et al. Prevalence of interstitial pneumonia suggestive of COVID-19 at (18)F-FDG PET/CT in oncological asymptomatic patients in a high prevalence country during pandemic period: a national multi-centric retrospective study. Eur J Nucl Med Mol Imaging. 2021. 10.1007/s00259-021-05219-0. 10.1007/s00259-021-05219-0 PMC7871520 33560453

[35] Wei WE, Li Z, Chiew CJ, Yong SE, Toh MP, Lee VJ (2020). Presymptomatic transmission of SARS-CoV-2—Singapore, January 23–March 16, 2020. MMWR Morb Mortal Wkly Rep.

[36] Polverari G, Arena V, Ceci F, Pelosi E, Ianniello A, Poli E (2020). (18)F-Fluorodeoxyglucose uptake in patient with asymptomatic severe acute respiratory syndrome coronavirus 2 (coronavirus disease 2019) referred to positron emission tomography/computed tomography for NSCLC restaging. J Thorac Oncol.

[37] Papa A, Pozzessere C, Cicone F, Rizzuto F, Cascini GL (2020). Not all that glitters is COVID! Differential diagnosis of FDG-avid interstitial lung disease in low-prevalence regions. Eur J Hybrid Imaging.

[38] Jajodia A, Ebner L, Heidinger B, Chaturvedi A, Prosch H (2020). Imaging in corona virus disease 2019 (COVID-19)—a scoping review. Eur J Radiol Open.

[39] Bahloul A, Boursier C, Jeulin H, Imbert L, Mandry D, Karcher G (2021). CT abnormalities evocative of lung infection are associated with lower (18)F-FDG uptake in confirmed COVID-19 patients. Eur J Nucl Med Mol Imaging.

[40] Bai HX, Hsieh B, Xiong Z, Halsey K, Choi JW, Tran TML (2020). Performance of radiologists in differentiating COVID-19 from non-COVID-19 viral pneumonia at chest CT. Radiology..

[41] Artigas C, Lemort M, Mestrez F, Gil T, Flamen P (2020). COVID-19 pneumonia mimicking immunotherapy-induced pneumonitis on 18F-FDG PET/CT in a patient under treatment with Nivolumab. Clin Nucl Med.

[42] Stasiak CES, Cardoso FR, de Almeida SA, Rosado-de-Castro PH (2021). Incidental finding of COVID-19 infection after [(68)Ga]Ga-PSMA-11 PET/CT imaging in a patient with prostate cancer. Eur J Nucl Med Mol Imaging.

[43] Olivari L, Riccardi N, Rodari P, Angheben A, Artioli P, Salgarello M (2020). COVID-19 pneumonia: increased choline uptake with 18F-choline PET/CT. Eur J Nucl Med Mol Imaging.

[44] Scarlattei M, Baldari G, Silva M, Bola S, Sammartano A, Migliari S (2020). Unknown SARS-CoV-2 pneumonia detected by PET/CT in patients with cancer. Tumori..

[45] de Galiza BF, Queiroz MA, Nunes RF, Costa LB, Zaniboni EC, Marin JFG (2020). Nonprostatic diseases on PSMA PET imaging: a spectrum of benign and malignant findings. Cancer Imaging.

[46] Savelli G, Bonacina M, Rizzo A, Zaniboni A (2020). Activated macrophages are the main inflammatory cell in COVID-19 interstitial pneumonia infiltrates. Is it possible to show their metabolic activity and thus the grade of inflammatory burden with (18)F-Fluorocholine PET/CT?. Med Hypotheses.

[47] Müller C, Schibli R, Maurer B (2020). Can nuclear imaging of activated macrophages with folic acid-based radiotracers serve as a prognostic means to identify COVID-19 patients at risk?. Pharmaceuticals..

[48] Scimeca M, Urbano N, Bonfiglio R, Montanaro M, Bonanno E, Schillaci O et al. Imaging diagnostics and pathology in SARS-CoV-2-related diseases. Int J Mol Sci. 2020;21(18). 10.3390/ijms21186960. 10.3390/ijms21186960 PMC7554796 32971906

[49] Qin C, Liu F, Yen TC, Lan X (2020). (18)F-FDG PET/CT findings of COVID-19: a series of four highly suspected cases. Eur J Nucl Med Mol Imaging.

[50] Zou S, Zhu X (2020). FDG PET/CT of COVID-19. Radiology..

[51] Rafiee F, Keshavarz P, Katal S, Assadi M, Nejati SF, Sadabad FE et al., editors. Coronavirus disease 2019 (COVID-19) in molecular imaging: a systematic review of incidental detection of SARS-CoV-2 pneumonia on PET studies. Semin Nucl Med. 2020. Elsevier. 10.1053/j.semnuclmed.2020.10.002 PMC7598766 33509374

[52] Dietz M, Chironi G, Claessens YE, Farhad RL, Rouquette I, Serrano B (2021). COVID-19 pneumonia: relationship between inflammation assessed by whole-body FDG PET/CT and short-term clinical outcome. Eur J Nucl Med Mol Imaging.

[53] Minamimoto R, Hotta M, Ishikane M, Inagaki T (2020). FDG-PET/CT images of COVID-19: a comprehensive review. Glob Health Med.

[54] Association CM (2020). Radiological diagnosis of new coronavirus infected pneumonitis: expert recommendation from the Chinese Society of Radiology. Chin J Radiol.

[55] Chung M, Bernheim A, Mei X, Zhang N, Huang M, Zeng X (2020). CT imaging features of 2019 novel coronavirus (2019-nCoV). Radiology..

[56] Chefer S, Thomasson D, Seidel J, Reba RC, Bohannon JK, Lackemeyer MG (2015). Modeling [(18)F]-FDG lymphoid tissue kinetics to characterize nonhuman primate immune response to Middle East respiratory syndrome-coronavirus aerosol challenge. EJNMMI Res.

[57] Sardanelli F, Cozzi A, Monfardini L, Bna C, Foa RA, Spinazzola A (2020). Association of mediastinal lymphadenopathy with COVID-19 prognosis. Lancet Infect Dis.

[58] Valette X, du Cheyron D, Goursaud S (2020). Mediastinal lymphadenopathy in patients with severe COVID-19. Lancet Infect Dis.

[59] O'Neill H, Doran S, Fraioli F, Nasoodi A (2020). A twisted tale-radiological imaging features of COVID-19 on (18)F-FDG PET/CT. Eur J Hybrid Imaging..

[60] Chadburn A, Metroka C, Mouradian J (1989). Progressive lymph node histology and its prognostic value in patients with acquired immunodeficiency syndrome and AIDS-related complex. Hum Pathol.

[61] Fu C, Zhang W, Li H, Bai Y, Bae KT, Wang M (2020). FDG PET/CT evaluation of a patient recovering from COVID-19. Eur J Nucl Med Mol Imaging.

[62] Steinberg J, Thomas A, Iravani A. 18Fluorodeoxyglucose PET/CT findings in a systemic inflammatory response syndrome after COVID-19 vaccine. Lancet. 2021. 10.1016/S0140-6736(21)00464-5 PMC7972305 33705696

[63] Özütemiz C, Krystosek LA, Church AL, Chauhan A, Ellermann JM, Domingo-Musibay E et al. Lymphadenopathy in COVID-19 vaccine recipients: diagnostic dilemma in oncology patients. Radiology. 2021;210275. 10.1148/radiol.2021210275 PMC7909072 33625300

[64] Wang D, Hu B, Hu C, Zhu F, Liu X, Zhang J (2020). Clinical characteristics of 138 hospitalized patients with 2019 novel coronavirus–infected pneumonia in Wuhan, China. Jama.

[65] Huang C, Wang Y, Li X, Ren L, Zhao J, Hu Y (2020). Clinical features of patients infected with 2019 novel coronavirus in Wuhan, China. Lancet.

[66] Tzotzos SJ, Fischer B, Fischer H, Zeitlinger M (2020). Incidence of ARDS and outcomes in hospitalized patients with COVID-19: a global literature survey. Crit Care.

[67] Boyle AJ, Mac Sweeney R, McAuley DF (2013). Pharmacological treatments in ARDS: a state-of-the-art update. BMC Med.

[68] Bellani G, Messa C, Guerra L, Spagnolli E, Foti G, Patroniti N (2009). Lungs of patients with acute respiratory distress syndrome show diffuse inflammation in normally aerated regions: a [18F]-fluoro-2-deoxy-D-glucose PET/CT study. Crit Care Med.

[69] Rodrigues RS, Miller PR, Bozza FA, Marchiori E, Zimmerman GA, Hoffman JM (2008). FDG-PET in patients at risk for acute respiratory distress syndrome: a preliminary report. Intensive Care Med.

[70] Rodrigues RS, Bozza FA, Hanrahan CJ, Wang LM, Wu Q, Hoffman JM (2017). (18)F-fluoro-2-deoxyglucose PET informs neutrophil accumulation and activation in lipopolysaccharide-induced acute lung injury. Nucl Med Biol.

[71] Merad M, Martin JC (2020). Pathological inflammation in patients with COVID-19: a key role for monocytes and macrophages. Nat Rev Immunol.

[72] Muller C, Schibli R, Maurer B. Can nuclear imaging of activated macrophages with folic acid-based radiotracers serve as a prognostic means to identify COVID-19 patients at risk? Pharmaceuticals (Basel). 2020;13(9). 10.3390/ph13090238. 10.3390/ph13090238 PMC7559490 32916949

[73] Manna S, Wruble J, Maron SZ, Toussie D, Voutsinas N, Finkelstein M (2020). COVID-19: a multimodality review of radiologic techniques, clinical utility, and imaging features. Radiol Cardiothorac Imaging.

[74] Liao SC, Shao SC, Chen YT, Chen YC, Hung MJ (2020). Incidence and mortality of pulmonary embolism in COVID-19: a systematic review and meta-analysis. Crit Care.

[75] Rothberg MB, Haessler SD (2010). Complications of seasonal and pandemic influenza. Crit Care Med.

[76] Bikdeli B, Wang Y, Jimenez D, Parikh SA, Monreal M, Goldhaber SZ (2019). Pulmonary embolism hospitalization, readmission, and mortality rates in US older adults, 1999-2015. JAMA..

[77] Estrada YMRM, Oldham SA (2011). CTPA as the gold standard for the diagnosis of pulmonary embolism. Int J Comput Assist Radiol Surg.

[78] Burger IA, Niemann T, Patriki D, Fontana F, Beer JH (2020). Is there a role for lung perfusion [(99m)Tc]-MAA SPECT/CT to rule out pulmonary embolism in COVID-19 patients with contraindications for iodine contrast?. Eur J Nucl Med Mol Imaging.

[79] Ferrando-Castagnetto F, Wakfie-Corieh CG, Garcia AMB, Garcia-Esquinas MG, Caro RMC, Delgado JLC (2020). Incidental and simultaneous finding of pulmonary thrombus and COVID-19 pneumonia in a cancer patient derived to (18)F-FDG PET/CT. New pathophysiological insights from hybrid imaging. Radiol Case Rep.

[80] Kaghazchi F, Borja A, Rojulpote C, Seraj SM, Zadeh MZ, Werner T (2020). Role of FDG-PET/CT in the detection and management of venous thromboembolism. J Nucl Med.

[81] Rondina MT, Lam UT, Pendleton RC, Kraiss LW, Wanner N, Zimmerman GA (2012). 18F-FDG PET in the evaluation of acuity of deep vein thrombosis. Clin Nucl Med.

[82] Hess S, Madsen PH, Iversen ED, Frifelt JJ, Hoilund-Carlsen PF, Alavi A (2015). Efficacy of FDG PET/CT imaging for venous thromboembolic disorders: preliminary results from a prospective, observational pilot study. Clin Nucl Med.

[83] Spagnolo P, Balestro E, Aliberti S, Cocconcelli E, Biondini D, Casa GD (2020). Pulmonary fibrosis secondary to COVID-19: a call to arms?. Lancet Respir Med.

[84] Shi H, Han X, Jiang N, Cao Y, Alwalid O, Gu J (2020). Radiological findings from 81 patients with COVID-19 pneumonia in Wuhan, China: a descriptive study. Lancet Infect Dis.

[85] Masclans JR, Roca O, Munoz X, Pallisa E, Torres F, Rello J (2011). Quality of life, pulmonary function, and tomographic scan abnormalities after ARDS. Chest..

[86] Herridge MS, Tansey CM, Matte A, Tomlinson G, Diaz-Granados N, Cooper A (2011). Functional disability 5 years after acute respiratory distress syndrome. N Engl J Med.

[87] Desogere P, Tapias LF, Hariri LP, Rotile NJ, Rietz TA, Probst CK et al. Type I collagen-targeted PET probe for pulmonary fibrosis detection and staging in preclinical models. Sci Transl Med. 2017;9(384). 10.1126/scitranslmed.aaf4696. 10.1126/scitranslmed.aaf4696 PMC5568793 28381537

[88] Munger JS, Huang XZ, Kawakatsu H, Griffiths MJD, Dalton SL, Wu JF (1999). The integrin alpha v beta 6 binds and activates latent TGF beta 1: a mechanism for regulating pulmonary inflammation and fibrosis. Cell.

[89] Montesi SB, Izquierdo-Garcia D, Desogere P, Abston E, Liang LL, Digumarthy S (2019). Type I collagen-targeted positron emission tomography imaging in idiopathic pulmonary fibrosis: first-in-human studies. Am J Respir Crit Care Med.

[90] Lukey PT, Coello C, Gunn R, Parker C, Wilson FJ, Saleem A (2020). Clinical quantification of the integrin alphavbeta6 by [(18)F]FB-A20FMDV2 positron emission tomography in healthy and fibrotic human lung (PETAL Study). Eur J Nucl Med Mol Imaging.

[91] Foster CC, Davis RA, Hausner SH, Sutcliffe JL (2020). alphavbeta6-targeted molecular PET/CT imaging of the lungs after SARS-CoV-2 infection. J Nucl Med.

[92] Behzad S, Aghaghazvini L, Radmard AR, Gholamrezanezhad A (2020). Extrapulmonary manifestations of COVID-19: radiologic and clinical overview. Clin Imaging.

[93] Mao L, Jin H, Wang M, Hu Y, Chen S, He Q (2020). Neurologic manifestations of hospitalized patients with coronavirus disease 2019 in Wuhan, China. JAMA Neurol.

[94] Egbert AR, Cankurtaran S, Karpiak S (2020). Brain abnormalities in COVID-19 acute/subacute phase: a rapid systematic review. Brain Behav Immun.

[95] Rogers JP, Chesney E, Oliver D, Pollak TA, McGuire P, Fusar-Poli P (2020). Psychiatric and neuropsychiatric presentations associated with severe coronavirus infections: a systematic review and meta-analysis with comparison to the COVID-19 pandemic. Lancet Psychiatry.

[96] Bohmwald K, Galvez NMS, Rios M, Kalergis AM (2018). Neurologic alterations due to respiratory virus infections. Front Cell Neurosci.

[97] Guedj E, Million M, Dudouet P, Tissot-Dupont H, Bregeon F, Cammilleri S (2021). (18)F-FDG brain PET hypometabolism in post-SARS-CoV-2 infection: substrate for persistent/delayed disorders?. Eur J Nucl Med Mol Imaging.

[98] Meinhardt J, Radke J, Dittmayer C, Franz J, Thomas C, Mothes R (2021). Olfactory transmucosal SARS-CoV-2 invasion as a port of central nervous system entry in individuals with COVID-19. Nat Neurosci.

[99] Delorme C, Paccoud O, Kas A, Hesters A, Bombois S, Shambrook P (2020). COVID-19-related encephalopathy: a case series with brain FDG-positron-emission tomography/computed tomography findings. Eur J Neurol.

[100] Grimaldi S, Lagarde S, Harle JR, Boucraut J, Guedj E (2020). Autoimmune encephalitis concomitant with SARS-CoV-2 infection: insight from (18)F-FDG PET imaging and neuronal autoantibodies. J Nucl Med.

[101] Li W, Yang Y, Liu ZH, Zhao YJ, Zhang Q, Zhang L (2020). Progression of mental health services during the COVID-19 outbreak in China. Int J Biol Sci.

[102] Mamun MA, Ullah I. COVID-19 suicides in Pakistan, dying off not COVID-19 fear but poverty?—the forthcoming economic challenges for a developing country. Brain Behav Immun. 2020. 10.1016/j.bbi.2020.05.028 PMC7212955 32407859

[103] Morbelli S, Ekmekcioglu O, Barthel H, Albert NL, Boellaard R, Cecchin D, et al. COVID-19 and the brain: impact on nuclear medicine in neurology: Springer; 2020. 10.1007/s00259-020-04965-x PMC7375837 32700058

[104] Nobili F, Arbizu J, Bouwman F, Drzezga A, Agosta F, Nestor P (2018). European Association of Nuclear Medicine and European Academy of Neurology recommendations for the use of brain 18F-fluorodeoxyglucose positron emission tomography in neurodegenerative cognitive impairment and dementia: Delphi consensus. Eur J Neurol.

[105] Zheng YY, Ma YT, Zhang JY, Xie X (2020). COVID-19 and the cardiovascular system. Nat Rev Cardiol.

[106] Zhou F, Yu T, Du R, Fan G, Liu Y, Liu Z (2020). Clinical course and risk factors for mortality of adult inpatients with COVID-19 in Wuhan, China: a retrospective cohort study. Lancet..

[107] Driggin E, Madhavan MV, Bikdeli B, Chuich T, Laracy J, Biondi-Zoccai G (2020). Cardiovascular considerations for patients, health care workers, and health systems during the COVID-19 pandemic. J Am Coll Cardiol.

[108] Puntmann VO, Carerj ML, Wieters I, Fahim M, Arendt C, Hoffmann J (2020). Outcomes of cardiovascular magnetic resonance imaging in patients recently recovered from coronavirus disease 2019 (COVID-19). JAMA Cardiol.

[109] Zoghbi WA, DiCarli MF, Blankstein R, Choi AD, Dilsizian V, Flachskampf FA (2020). Multimodality cardiovascular imaging in the midst of the COVID-19 pandemic: ramping up safely to a new normal. JACC Cardiovasc Imaging.

[110] Sollini M, Ciccarelli M, Cecconi M, Aghemo A, Morelli P, Gelardi F, et al. Vasculitis changes in COVID-19 survivors with persistent symptoms: an [(18)F]FDG-PET/CT study. Eur J Nucl Med Mol Imaging. 2020. 10.1007/s00259-020-05084-3. 10.1007/s00259-020-05084-3 PMC7595761 33123760

[111] Halsey R, Priftakis D, Mackenzie S, Wan S, Davis LM, Lilburn D (2021). COVID-19 in the act: incidental 18F-FDG PET/CT findings in asymptomatic patients and those with symptoms not primarily correlated with COVID-19 during the United Kingdom coronavirus lockdown. Eur J Nucl Med Mol Imaging.

[112] Jin Z, Du X, Xu Y, Deng Y, Liu M, Zhao Y, et al. Structure-based drug design, virtual screening and high-throughput screening rapidly identify antiviral leads targeting COVID-19. BioRxiv. 2020.

[113] Willmann JK, van Bruggen N, Dinkelborg LM, Gambhir SS (2008). Molecular imaging in drug development. Nat Rev Drug Discov.

[114] Wang J, Maurer L (2005). Positron emission tomography: applications in drug discovery and drug development. Curr Top Med Chem.

[115] Neumaier F, Zlatopolskiy BD, Neumaier B. Nuclear medicine in times of COVID-19: how radiopharmaceuticals could help to fight the current and future pandemics. Pharmaceutics. 2020;12(12). 10.3390/pharmaceutics12121247. 10.3390/pharmaceutics12121247 PMC7767508 33371500

[116] Shiri I, Abdollahi H, Atashzar MR, Rahmim A, Zaidi H (2020). A theranostic approach based on radiolabeled antiviral drugs, antibodies and CRISPR-associated proteins for early detection and treatment of SARS-CoV-2 disease. Nucl Med Commun.

[117] Jalilian AR (2016). An overview on Ga-68 radiopharmaceuticals for positron emission tomography applications. Iran J Nucl Med.

[118] Qaim SM, Scholten B, Neumaier B (2018). New developments in the production of theranostic pairs of radionuclides. J Radioanal Nucl Chem.

[119] Rabaan AA, Al-Ahmed SH, Haque S, Sah R, Tiwari R, Malik YS (2020). SARS-CoV-2, SARS-CoV, and MERS-COV: a comparative overview. Infez Med.

[120] Lutje S, Marinova M, Kutting D, Attenberger U, Essler M, Bundschuh RA (2020). Nuclear medicine in SARS-CoV-2 pandemia: (18) F-FDG-PET/CT to visualize COVID-19. Nuklearmedizin-Nucl Med.

[121] Jafarzadeh A, Chauhan P, Saha B, Jafarzadeh S, Nemati M (2020). Contribution of monocytes and macrophages to the local tissue inflammation and cytokine storm in COVID-19: lessons from SARS and MERS, and potential therapeutic interventions. Life Sci.

[122] Gomez-Rial J, Rivero-Calle I, Salas A, Martinon-Torres F (2020). Role of monocytes/macrophages in Covid-19 pathogenesis: implications for therapy. Infect Drug Resist.

[123] Jiemy WF, Heeringa P, Kamps J, van der Laken CJ, Slart R, Brouwer E (2018). Positron emission tomography (PET) and single photon emission computed tomography (SPECT) imaging of macrophages in large vessel vasculitis: current status and future prospects. Autoimmun Rev.

